# Evaluating infection prevention and control programs in Austrian acute care hospitals using the WHO Infection Prevention and Control Assessment Framework

**DOI:** 10.1186/s13756-020-00761-2

**Published:** 2020-06-22

**Authors:** Seven Johannes Sam Aghdassi, Andrea Grisold, Agnes Wechsler-Fördös, Sonja Hansen, Peter Bischoff, Michael Behnke, Petra Gastmeier

**Affiliations:** 1 Charité – Universitätsmedizin Berlin, corporate member of Freie Universität Berlin, Humboldt-Universität zu Berlin, and Berlin Institute of Health, Institute of Hygiene and Environmental Medicine, Berlin, Germany; 2 National Reference Center for Surveillance of Nosocomial Infections, Berlin, Germany; 3 D&R Institute of Hygiene, Microbiology and Environmental Medicine, Medical University, Graz, Austria; 4 Austrian Society of Hygiene, Microbiology and Preventive Medicine, Vienna, Austria

**Keywords:** Infection prevention, Self-assessment, Survey, WHO, Austria

## Abstract

**Background:**

Infection prevention and control (IPC) is crucial for patient safety. The World Health Organization (WHO) has released various tools to promote IPC. In 2018, the WHO released the Infection Prevention and Control Assessment Framework (IPCAF) that enables acute care healthcare facilities to evaluate IPC structures and practices. Data regarding IPC implementation in Austria are scarce. To deliver insights into this topic and promote the IPCAF within the Austrian IPC community, we decided to invite all Austrian hospitals participating in the German nosocomial infection surveillance system to conduct a self-assessment using the WHO IPCAF.

**Methods:**

The IPCAF follows the eight WHO core components of IPC. A German translation of the IPCAF was sent to 127 Austrian acute care hospitals. The survey period was from October to December 2018. Participation in the survey, data entry and transfer to the German national reference center for surveillance of healthcare-associated infections was on a voluntary basis.

**Results:**

Altogether, 65 Austrian hospitals provided a complete dataset. The overall median IPCAF score of all hospitals was 620 (of a possible maximum score of 800), which corresponded to an advanced level of IPC. Of the 65 hospitals, 38 achieved an advanced IPC level. Deeper analysis of the different core components yielded diverse results. Scores were lowest for core components on multimodal strategies for implementation of IPC interventions, and IPC education and training. Around 26% (*n* = 17) of hospitals reported that the local IPC team was not steadily supported by an IPC committee. Senior clinical staff was not present in the IPC committee in 23% (*n* = 15) of hospitals. Only 26% (n = 17) of hospitals reported employing at least one IPC professional per ≤250 beds. Surveillance for multidrug-resistant pathogens was not conducted in 26% (n = 17) of hospitals.

**Conclusions:**

Implementation of IPC key aspects is generally at a high level in Austria. However, potentials for improvement were demonstrated, most prominently with regard to staffing, IPC education and training, effective implementation of multimodal strategies, and involvement of professional groups. Our survey demonstrated that the IPCAF is a useful tool for IPC self-assessment and can uncover deficits even in a high-income setting like Austria.

## Background

According to a national point prevalence survey on healthcare-associated infections (HAI) in Austria, the prevalence of patients with HAI in Austrian acute care hospitals is estimated to be around 4% [1]. Surveillance of HAI in Austria has historically been organized in various networks. Due to its extensiveness and the various modules it entails, many hospitals in Austria have decided to join the German national surveillance network “KISS” (*Krankenhaus-Infektions-Surveillance-System*). In the last two decades, steps have been undertaken to strengthen infection prevention and control (IPC) activities in Austria [2]. Efforts have been coordinated by the Austrian Federal Ministry of Labor, Social Affairs, Health and Consumer Protection (*Bundesministerium für Arbeit, Soziales, Gesundheit und Konsumentenschutz*) to commit hospitals to conducting surveillance and reporting data on certain indicator infections to the ministry. These measures serve the goal of establishing a database for national HAI reference data. IPC key aspects additional to HAI surveillance, however, have not been investigated systematically in Austria yet.

The World Health Organization (WHO) offers a variety of guideline documents and tools, which enable healthcare facilities to evaluate certain IPC processes and structures [3–6]. In an attempt to release an overarching tool that addresses not only selected IPC aspects, but infection control in its entire complexity, the WHO released the Infection Prevention and Control Assessment Framework (IPCAF) in 2018 [7].

Due to the novelty of the IPCAF, data on results derived from its application are scarce. In a previous publication, we described the use of the IPCAF in the context of a national survey in Germany [8]. The survey demonstrated that IPC is generally at a high level in German acute care hospitals, particularly with regard to HAI surveillance. Nevertheless, certain sections of the IPCAF, namely on multimodal strategies for implementation of IPC interventions and staffing revealed considerable potentials for improvement.

Both Austria and Germany are high-income countries and share many cultural and infrastructural similarities, also with regard to healthcare and public health [9]. It was therefore our objective to describe the state of implementation of IPC aspects in Austrian hospitals participating in the German national surveillance network, and where appropriate, compare results from Austrian hospitals to data from the survey conducted in Germany. Furthermore, it was our goal to increase awareness of WHO recommendations for IPC among Austrian healthcare professionals.

## Methods

The German National Reference Center for Surveillance of Nosocomial Infections (*Nationales Referenzzentrum für Surveillance von nosokomialen Infektionen*, NRZ) organizes surveillance activities in Germany and has created the KISS network. In the year 2018, 1297 hospitals and other healthcare facilities actively participated (i.e. had entered data into the KISS database) in the network, 105 of which were from Austria. In order to investigate IPC aspects beyond surveillance, surveys on different topics are sent to participating hospitals on a regular basis. In 2018, the recently released WHO IPCAF was chosen as a suitable survey. In order to minimize the potential language barrier, the NRZ translated the IPCAF into German. The translated version of the IPCAF can be found in the online supplement of this article (additional file 1). On the first of October 2018, the IPCAF was sent to 127 acute care hospitals in Austria that had participated in the KISS network at any point in time. Data entry was possible until the end of the year. During the survey period, one email reminder was sent in the beginning of December 2018. Participation was on a voluntary basis. The specifics of survey organization were described in more detail in a previous publication [8]. To comply with data protection regulations, no linkage of survey data with other structural and process parameters, or data on HAI was undertaken. However, structural characteristics of the 127 hospitals invited are illustrated in table e1 of the online supplement (additional file 2).

The IPCAF is divided into eight sections, which reflect the eight WHO “Core Components of Infection Prevention and Control Programmes” [3]. These are:

- Core component (CC) 1: IPC Program
- CC2: IPC Guidelines
- CC3: IPC Education and Training
- CC4: HAI Surveillance
- CC5: Multimodal Strategies for Implementation of IPC Interventions
- CC6: Monitoring/Audit of IPC Practices and Feedback
- CC7: Workload, Staffing and Bed Occupancy
- CC8: Environments, Materials and Equipment for IPC

The tool follows a questionnaire-like format and is designed to enable healthcare facilities from all healthcare settings and income levels to perform an IPC self-assessment with the goal of identifying deficits and potentials for improvement. Per CC, a maximum score of 100 points can be achieved, hence the highest possible overall IPCAF score is 800 points. Depending on the overall score, an IPC level is allocated to a healthcare facility. Scores from 0 to 200 points correspond to an inadequate, 201–400 points to a basic, 401–600 points to an intermediate, and 601–800 points to an advanced IPC level.

Since the survey was organized by the German NRZ, data management was in alignment with German laws. More precisely, all data were collected, anonymized and analyzed in accordance with the German Protection against Infection Act, which regulates the prevention and control of infectious diseases in humans, and also addresses surveillance of HAI and IPC in general [10]. Ethical approval and informed consent were therefore not required.

## Results

**Fig. 1:**
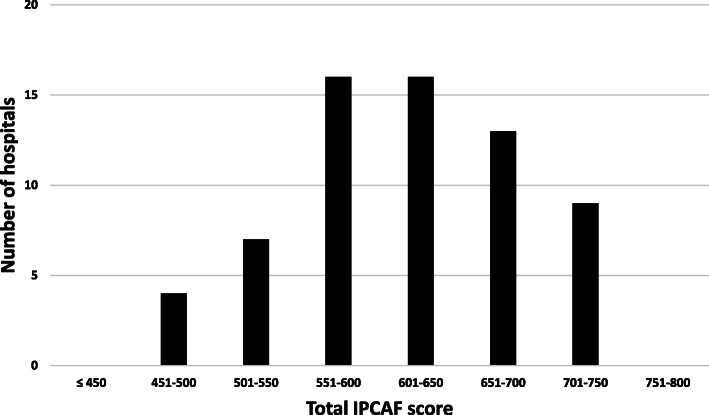
Distribution of the total IPCAF score among participating hospitals. Results from 65 participating Austrian acute care hospitals. **Legend** Fig. 1: Abbreviations: *IPCAF* Infection Prevention and Control Assessment Framework

Of the 127 acute care hospitals invited, 65 participated and provided a complete dataset (response rate of 51%). No incomplete datasets were received. As a net result of all participating hospitals, a median score of 620 was achieved, with an interquartile range from 568 to 675. When looking at the individual scores of hospitals, points ranged from 458 to 743 (Fig. 1). When applying the IPC level allocation as suggested by the WHO, 27 hospitals (42%) fell into the category “intermediate”, and 38 (58%) achieved a score that corresponded to an “advanced” IPC level. In no cases, an “inadequate” or “basic” IPC level was allocated.

Profound differences were revealed with regard to the scores of the individual components of the IPCAF. Table 1 illustrates the tenth, 25th, 50th, 75th, and 90th percentile, as well as the mean, for the IPCAF net score and for scores of the individual CC. The lowest median score (65) was recorded for CC5 (Multimodal Strategies for Implementation of IPC Interventions), while the highest scores were recorded for CC8 (Environments, Materials and Equipment for IPC).

**Table 1:** Distribution of results of the total IPCAF score and scores per core component. Results from 65 participating Austrian acute care hospitals

Component	Score					
	P10	Q1	Median	Q3	P90	Mean
CC1: IPC Program	57.5	62.5	77.5	85	91.5	74.3
CC2: IPC Guidelines	77.5	87.5	95	97.5	100	91.5
CC3: IPC Education and Training	50	65	70	75	85	69.4
CC4: HAI Surveillance	58.5	70	82.5	90	92.5	78.4
CC5: Multimodal Strategies for Implementation of IPC Interventions	6	40	65	80	88	57.6
CC6: Monitoring/Audit of IPC Practices and Feedback	53.5	62.5	72.5	80	89	72.1
CC7: Workload, Staffing and Bed Occupancy	52	65	80	95	98	77.2
CC8: Environments, Materials and Equipment for IPC	90	95	95	100	100	95.7
Total	513.5	567.5	620	675	709	616.2

Abbreviations: *CC* Core component(s); *HAI* Healthcare-associated infection(s); *IPCAF* Infection Prevention and Control Assessment Framework; *P10* Tenth percentile; *P90* 90th percentile; *Q1* First quartile; *Q3* Third quartile

Concerning CC1 (IPC Program), only 26% of hospitals (*n* = 17) reported employing at least one IPC professional per ≤250 beds. Three hospitals (5%) reported not having an IPC program in place. Of those that reported having such a program, around half (*n* = 30) stated that the program included clearly defined objectives, while the other half (*n* = 32) reported that these were lacking. A more detailed question regarding IPC objectives revealed that measureable outcome indicators and set future targets were part of IPC objectives in 10 hospitals (15%), with another 21 hospitals (32%) having at least measureable outcome indicators in place, but no set future targets. With regard to CC7 (Workload, Staffing and Bed Occupancy), it was reported that 40% of hospitals (*n* = 26) did not ensure an agreed healthcare worker (i.e. personnel directly involved in patient care) to patient ratio at all times within the entire facility. Surveillance of multidrug-resistant pathogens according to the local epidemiological situation was reportedly not conducted in 17 hospitals (26%). Further results of particular relevance and interest from other sections of the IPCAF are listed in Table 2.

**Table 2:** Selected results from various core components of the IPCAF. Results from 65 participating Austrian acute care hospitals

Topic	Answer	Number (%)
Staffing with IPC professionals (CC1: IPC Program)	No IPC professional	0 (0)
	Only part-time IPC professional	16 (24.6)
	One IPC professional per > 250 beds	39 (49.2)
	One IPC professional per ≤250 beds	17 (26.2)
Support from IPC committee (CC1: IPC Program)	IPC committee not established or not actively supporting IPC team	17 (26.2)
	IPC committee established and actively supporting IPC team	48 (73.8)
Professional groups in IPC committee – Senior facility leadership (CC1: IPC Program)	Not part of IPC committee	14 (21.5)
	Part of IPC committee	51 (78.5)
Professional groups in IPC committee – Senior clinical staff (CC1: IPC Program)	Not part of IPC committee	15 (23.1)
	Part of IPC committee	50 (76.9)
Professional groups in the IPC committee – Facility management (CC1: IPC Program)	Not part of IPC committee	28 (43.1)
	Part of IPC committee	37 (56.9)
Surveillance of multidrug-resistant pathogens according to local situation (CC4: HAI Surveillance)	Not conducted	17 (26.2)
	Conducted	48 (73.8)
Surveillance of infections in vulnerable populations (CC4: HAI Surveillance)	Not conducted	24 (36.9)
	Conducted	41 (63.1)
Analysis of antimicrobial drug resistance (CC4: HAI Surveillance)	Not or only irregularly conducted	21 (32.3)
	Regularly conducted	44 (67.7)
Feedback of surveillance data to frontline HCW (CC4: HAI Surveillance)	Not or only irregularly done	17 (26.2)
	Regularly done	48 (73.8)
Feedback of surveillance data to heads of departments (CC4: HAI Surveillance)	Not or only irregularly done	9 (13.8)
	Regularly done	56 (86.2)
Method of feedback of surveillance data (CC4: HAI Surveillance)	No annual feedback	4 (6.2)
	Annual feedback in written and/or oral form only	42 (64.6)
	Annual feedback via presentation and interactive problem-solution finding	19 (29.2)
Frequency of undertaking the WHO Hand Hygiene Self-Assessment Framework (CC6: Monitoring/Audit of IPC Practices and Feedback)	Never	46 (70.8)
	Irregularly	14 (21.5)
	Regularly (at least annually)	5 (7.7)

Abbreviations: *CC* Core component(s); *HAI* Healthcare-associated infection(s); *HCW* Healthcare worker(s); *IPC* Infection prevention and control; *IPCAF* Infection Prevention and Control Assessment Framework

Overall, 77% of hospitals (*n* = 50) reported employing multimodal strategies when implementing IPC interventions (CC5: Multimodal Strategies for Implementation of IPC Interventions). However, teams in charge of multimodal strategies were multidisciplinary in only 58% of hospitals (*n* = 38), and professionals working in quality improvement and patient safety were involved in only 66% of hospitals (*n* = 43). About 65% of hospitals (*n* = 42) reported utilizing bundles and checklists as components of their multimodal strategies. Table 3 illustrates results for the individual elements of multimodal strategies of CC5, as reported by participating Austrian hospitals.

**Table 3:** Results per element from Core Component 5 of the IPCAF: Multimodal Strategies for Implementation of Infection Prevention and Control Interventions. Results from 65 participating Austrian acute care hospitals

Element	Answer	Number (%)
System change	Element not included in multimodal strategies	18 (27.7)
	Interventions to ensure the necessary infrastructure and continuous availability of supplies are in place	18 (27.7)
	Interventions to ensure the necessary infrastructure and continuous availability of supplies are in place and addressing ergonomics and accessibility, such as the best placement of central venous catheter set and tray	29 (44.6)
Education and training	Element not included in multimodal strategies	10 (15.4)
	Written information and/or oral instruction and/or e-learning only	46 (70.8)
	Additional interactive training sessions (includes simulation and/or bedside training)	9 (13.8)
Monitoring and feedback	Element not included in multimodal strategies	18 (27.7)
	Monitoring compliance with process or outcome indicators (for example, audits of hand hygiene or catheter practices)	28 (43.1)
	Monitoring compliance and providing timely feedback of monitoring results to health care workers and key players	19 (29.2)
Communications and reminders	Element not included in multimodal strategies	14 (21.5)
	Reminders, posters, or other advocacy/awareness-raising tools to promote the intervention	42 (64.6)
	Additional methods/initiatives to improve team communication across units and disciplines (for example, by establishing regular case conferences and feedback rounds)	9 (13.8)
Safety climate and culture change	Element not included in multimodal strategies	21 (32.3)
	Managers/leaders show visible support and act as champions and role models, promoting an adaptive approach and strengthening a culture that supports IPC, patient safety and quality	39 (60)
	Additionally, teams and individuals are empowered so that they perceive ownership of the intervention (for example, by participatory feedback rounds)	5 (7.7)

Abbreviations: *IPCAF* Infection Prevention and Control Assessment Framework

With regard to CC3 (IPC Education and Training), it was reported that almost all hospitals (98%) had expertise on site to perform IPC training. Additional non-IPC staff capable of training (e.g. link nurses or doctors) were reportedly present at 63% of hospitals (*n* = 41). Specifics of how training was conducted (i.e. frequency and didactic methods, as well as evaluation of effectiveness) are illustrated in Table 4. About half of participating hospitals (52%; *n* = 34) reported conducting specific IPC training for patients or family members (e.g. for targeted vulnerable patient populations) to involve them in preventive measures to minimize the risk for HAI. All hospitals reported offering their IPC teams the opportunity for continuous education (e.g. through attendance of conferences).

**Table 4:** Results on frequency, didactic methods and evaluation from Core Component 3 of the IPCAF: Infection Prevention and Control Education and Training. Results from 65 participating Austrian acute care hospitals

Topic	Answer	Number (%)
Frequency of IPC training for frontline HCW	Never or rarely	0 (0)
	Only for new employees	6 (9.2)
	For new employees, and at least annually for all HCW, but not mandatory	43 (66.2)
	For new employees, and at least annually mandatory training for all HCW	16 (24.6)
Frequency of IPC training for cleaners and other non-HCW personnel involved in patient care	Never or rarely	2 (3.1)
	Only for new employees	9 (13.8)
	For new employees, and at least annually for all HCW, but not mandatory	26 (40)
	For new employees, and at least annually mandatory training for all HCW	28 (43.1)
IPC training for administrative and managerial staff	Not established	32 (49.2)
	Established	33 (50.8)
Methods of IPC training for HCW and other staff	No training available	0 (0)
	Only in written and/or oral and/or online form	40 (61.5)
	Interactive training (e.g. bedside teaching)	25 (38.5)
Integration of IPC training into education of other medical disciplines	Not established	23 (35.4)
	Established, but not in all disciplines	29 (44.6)
	Established, in all disciplines	13 (20)
Frequency of evaluation of the effectiveness of IPC training programs	Never	21 (32.3)
	Irregular evaluation	23 (35.4)
	Regular evaluation	21 (32.3)

Abbreviations: *HCW* Healthcare worker(s); *IPC* Infection prevention and control; *IPCAF* Infection Prevention and Control Assessment Framework

Full survey results are available in the online supplement of this article (additional file 3).

## Discussion

Our survey has delivered valuable insights into the state of implementation of key IPC structures and processes in Austria. Overall, the data gathered demonstrated that IPC is at a high, yet in some respects diverse, level in Austria. This generally high level of IPC implementation was expected, as Austria is classified as a high-income country according to the World Bank classification [11]. Noticeably though, a considerable proportion of participating hospitals (42%) were allocated to merely an “intermediate” IPC level. This rather surprising finding could either be explained by a very strict interpretation of the IPCAF by Austrian hospitals, or be an indicator that IPC is not yet adequately addressed by some hospitals in Austria.

Besides the differences observed between the different participating Austrian hospitals with regard to the overall IPCAF score, we noticed pronounced differences between aggregated scores of the respective IPCAF sections. Scores regarding CC1 (IPC Program) were generally high. However, specific questions focusing on IPC staffing and organization of an IPC committee revealed mixed results. Only 26% of hospitals reported employing at least one IPC professional per ≤250 beds, which has to be viewed as a clear target for improvement. Moreover, more than one fourth of hospitals from Austria reported not having an IPC committee actively supporting the IPC team. Where IPC committees existed, crucial professional groups (e.g. senior facility leadership, senior clinical staff, and facility management) were underrepresented in many Austrian hospitals. In CC7 (Workload, Staffing and Bed Occupancy), only 60% of hospitals in Austria reported that an agreed healthcare worker to patient ratio was maintained at all times within the entire facility. Given the evidence, that understaffing is a demonstrated risk factor HAI occurrence [12], this finding gains relevance as an action point for future measures to improve the quality of care in Austrian hospitals.

A relatively high proportion (26%) of Austrian hospitals reported not conducting surveillance of multidrug-resistant pathogens according to the local epidemiological situation. Given the increase in awareness for the subject, due to recent publications on the burden of antimicrobial resistance in patients with HAI [13], we found this proportion to be surprisingly high. A crucial element of surveillance is feedback to relevant stakeholders, such as frontline healthcare workers and clinical leadership [14]. The majority of Austrian hospitals reported performing regular feedback of surveillance data. However, regarding the methods employed for this feedback, less than a third of participating hospitals reported performing annual feedback via presentation and interactive problem-solution finding. It is important to acknowledge this result as a target for improvement, since the effectiveness of interactive feedback of surveillance data has been demonstrated in previous publications [15, 16].

The concept of multimodal strategies has become increasingly prevalent in medicine, and in the practice of IPC [17–22]. However, given the rather low scores achieved by Austrian hospitals for CC5 (Multimodal Strategies for Implementation of IPC Interventions), it appears that awareness for and implementation of multimodal strategies are not yet fully accomplished. A similar deficit was illustrated in an analysis of the German IPCAF data [8]. A conclusion, which could be derived from these data, is that any efforts to strengthen IPC in high-income countries, should place an emphasis on multimodal strategies.

According to data from this survey, Austrian hospitals do not lack on-site expertise for conducting IPC training, as almost all hospitals reported having staff capable of performing IPC training. However, questions focusing on the execution of IPC training revealed deficits, especially with regard to the frequency of training and the methods employed. For instance, the percentage of hospitals that reported conducting mandatory annual training was lower than in the survey in Germany [8], where such activities are mandatory in numerous federal states. The importance of regular IPC education and training has been demonstrated in various publications [23], and given the presence of capable staff on-site, appears feasible in Austrian hospitals. Regarding methods employed for IPC training, similar shortcomings were demonstrated as in the German survey [8]. The lack of interactive (e.g. bedside) training has to be considered as a deficit and potential for improvement, as this form of education has been proven to be efficacious in numerous studies [20, 24].

A secondary objective of our survey was to increase the awareness of and knowledge about WHO tools in Austria. Our data suggests that these are not yet fully implemented in the routine IPC work at Austrian hospitals. For instance, only a minority of hospitals reported regularly conducting the WHO Hand Hygiene Self-Assessment Framework, despite the tool already having been released by the WHO in 2010 [25], and having been promoted in two global surveys (2011 and 2015) [26]. This finding may be attributable to the fact that the Austrian “Clean Care is Safer Care” campaign has been initiated only in recent years.

The results of our survey gain additional relevance when comparing the Austrian data to data from the IPCAF survey conducted in German hospitals. In Germany, the proportion of hospitals not achieving an advanced IPC level was only 15% (versus 42% in Austria). Moreover, the median aggregated IPCAF score was higher in Germany (690 in Germany vs. 620 in Austria) [8]. This discrepancy between the two economically and culturally similar countries, may partially be attributable to measures strengthening IPC in German hospitals which have been undertaken earlier in Germany than in Austria. The revision of the German Protection against Infection Act in 2011 required hospitals to reinforce existing IPC structures [10], particularly regarding IPC staffing and surveillance of HAI. It is conceivable that this has led to a more equal state of implementation of IPC in Germany. As of now, Austria does not have a similar legislation in place with regard to infection prevention in order to promote adequate staffing for IPC. Additionally, the long history of surveillance in Germany [27, 28], has to be recognized as factor contributing to high awareness of IPC in Germany.

Adequate interpretation of the data presented in this publication requires acknowledgement of various limitations. The primary purpose of the IPCAF is to enable healthcare facilities to perform a self-assessment of the local situation regarding IPC, not to generate national reference data. Comparisons between countries and healthcare systems are therefore only possible with significant restrictions. The NRZ is responsible for organizing surveillance in Germany, and the KISS network is a German institution. Therefore, collection of data from other countries is not the primary purpose of a survey issued by the NRZ. However, due to the large number of hospitals from Austria participating in the KISS network (approximately 40% of all acute care hospitals in Austria) and in this survey (around 25% of all acute care hospitals in Austria) [29], careful extrapolations to the overall situation in Austrian acute care hospitals appear justified. Nevertheless, it has to be noted that survey participation was on voluntary basis, therefore, facilities with a high interest in IPC may be overrepresented. This could lead to an overestimation of the state of IPC implementation in Austrian hospitals.

Proper utilization of the IPCAF required a deep understanding of the WHO terminology and underlying concepts. Despite providing footnotes and online support, concepts such as multimodal strategies, may have been misunderstood, and therefore certain data not reported correctly. Furthermore, selected questions of the IPCAF may have been deemed as compromising by some hospitals, which could lead to biased reporting in some cases. However, to decrease the incentive for wishful reporting and to comply with data protection restrictions, the IPCAF survey did not collect structural hospital information (e.g. number of beds, hospital type, and hospital ownership). Regarding data collection, it has to be acknowledged that the web-based mode of data entry did not allow for retroactively correcting entered data after completion of the survey. This may explain some surprising results.

## Conclusion

Overall, IPC structures and processes are at a relatively high level in Austria, especially with regard to IPC guidelines and certain aspects of HAI surveillance. Potentials for improvement were seen particularly with regard to multimodal strategies for implementation of IPC interventions, IPC education and training, staffing with IPC professionals and other healthcare workers, as well as involvement of relevant stakeholders. A comparison with data from a national survey in Germany using the IPCAF revealed many similarities. Overall implementation of IPC structures and processes in Austria was found to be at a slightly lower level than in Germany. Alike the survey conducted in Germany, the data gathered from Austrian hospitals showed that the IPCAF is a useful tool to evaluate IPC standards and uncover deficits even in high-income settings. Repeated application of the IPCAF should be encouraged in all healthcare settings to observe changes and trends and develop tailor-made strategies at promoting IPC.

## Supplementary information

**Additional file 1.** Infection Prevention and Control Assessment Framework (IPCAF) German translation.

**Additional file 2: Table e1.** Structural characteristics of 127 Austrian acute care hospitals invited to participate in the WHO Infection Prevention and Control Assessment Framework (IPCAF).

**Additional file 3.** Full results of the Infection Prevention and Control Assessment Framework (IPCAF) in 65 Austrian hospitals.

## Data Availability

The datasets used and analyzed in the context of this survey are available from the corresponding author upon reasonable request.

## Abbreviations

CC: Core component(s)
HAI: Healthcare-associated infection(s)
IPC: Infection prevention and control
IPCAF: Infection prevention and control assessment framework
KISS: Krankenhaus-infektions-surveillance-system
NRZ: National reference center for surveillance of nosocomial infections
WHO: World health organization
