# Supplementary material for: Evaluating infection prevention and control programs in Austrian acute care hospitals using the WHO Infection Prevention and Control Assessment Framework
Source: Antimicrob Resist Infect Control. 2020 Jun 22;9:92. doi: 10.1186/s13756-020-00761-2 (PMC7309981; doi:10.1186/s13756-020-00761-2)
Supplement: Supplementary file 1 — Additional file 1. Infection Prevention and Control Assessment Framework (IPCAF) German translation. [file 13756_2020_761_MOESM1_ESM.pdf]

Infection Prevention and Control Assessment Framework (IPCAF)

Instrument zur Bewertung der Hygiene und Infektionsprävention in Einrichtungen des Gesundheitswesens

Einleitung und Hinweise zur Anwendung des IPCAF

Das „**I**nfection **P**revention and **C**ontrol (IPC) **A**ssessment **F**ramework” (IPCAF) ist ein Erhebungsinstrument, welches die Implementierung von Empfehlungen der Weltgesundheitsorganisation (WHO) hinsichtlich wichtiger Kernkomponenten der „**I**nfection **P**revention and **C**ontrol“ (IPC)-Programme entsprechend der WHO-Leitlinien[1] unterstützt.
Nutzer des IPCAF sollten sich vor Anwendung des IPCAF mit den Inhalten der genannten WHO-Leitlinien sowie der dazugehörigen Anleitung zur Implementierung der WHO-Leitlinien[2], vertraut machen. Das IPCAF-Tool ermöglicht eine systematische Bewertung der Hygienestrukturen und Maßnahmen der Infektionsprävention in Krankenhäusern/Gesundheitseinrichtungen. Regelmäßig wiederholte Erhebungen mithilfe des IPCAF-Tools erlauben darüber hinaus eine Verlaufskontrolle sowie eine periodische Evaluierung von hygienerelevanten Strukturen und Aktivitäten.

Was ist der Zweck des IPCAF?

Das IPCAF ist ein Fragebogen mit fester Struktur aus geschlossenen Fragen, der mithilfe des dazugehörigen Punktesystems eigenständig ausgewertet werden kann. Das IPCAF ist als Selbstbeurteilungsbogen konzipiert. Somit können Krankenhäuser und andere Gesundheitseinrichtungen das Tool nutzen, um ihre Hygienestrukturen und Maßnahmen im Bereich der Infektionsprävention selbst beurteilen und einschätzen zu können. Das IPCAF eignet sich aber auch für Fremdbeurteilungen durch externe Gutachter (z.B. Mitarbeiter des Gesundheitsministeriums, der WHO oder anderer Interessensgruppen). In diesem Fall sollten die Beurteilungen jedoch in enger Kooperation zwischen den externen Gutachtern und dem hausinternen Fachpersonal erstellt und die Ergebnisse gemeinsam diskutiert und interpretiert werden.
Das IPCAF ist in erster Linie für Akutkrankenhäuser konzipiert, kann jedoch auch in anderen Einrichtungen eingesetzt werden. Das Tool wurde für den weltweiten Einsatz entwickelt und lässt sich sowohl auf einkommensstarke als auch einkommensschwache Länder oder Regionen gleichermaßen anwenden.

Ziel des IPCAF ist es, den Ist-Zustand im Hinblick auf bestehende Hygienestrukturen und Maßnahmen zur Infektionsprävention in der eigenen Einrichtung zu beurteilen. Diese Beurteilung lässt Rückschlüsse auf Stärken und Schwachstellen im aktuellen Hygiene- und Infektionspräventionsmanagement zu, die dann in zukünftigen Plänen berücksichtigt werden sollten. Das IPCAF kann als eine Art „Diagnosetool“ angesehen werden, welches relevante Probleme und Mängel aufzeigen kann, die verbessert werden sollten. Ebenso eignet es sich zur Identifikation von Bereichen, in denen internationale Standards und Anforderungen erreicht werden.
Bei Verwendung des IPCAF als Instrument zur Selbsteinschätzung ist es dabei erforderlich, die Fragen objektiv und korrekt zu beantworten, um eine effektive Selbsteinschätzung zu ermöglichen. Werden durch das IPCAF Anstrengungen und Verbesserungen im Prozess sichtbar gemacht, kann dies helfen Entscheidungsträger (z.B. Krankenhausträgerschaft) überzeugen, dass geeignete Maßnahmen zum Erfolg führen und Fortschritte bringen können. Die Identifikation von Schwachstellen kann wiederum alle Beteiligten dafür sensibilisieren, dass Veränderungen dringend notwendig sind. Diese

---

[1] WHO Guidelines on core components of IPC programmes at the national and acute health care facility level. 2016 (http://www.who.int/infection-prevention/publications/core-components/en/, Zugriff am 06. August 2018).
[2] Interim practical manual supporting implementation of the WHO guidelines on core components of infection prevention and control programmes. (http://www.who.int/infection-prevention/tools/core-components/en/, Zugriff am 06. August 2018)

Sensibilisierung ist eine wichtige Voraussetzung für Verbesserungen im Bereich der Hygiene und Infektionsprävention.
Um dies korrekt durchführen zu können, muss für jeden Abschnitt (Kernkomponenten 1-8) und für den gesamten Fragebogen die jeweils korrekte Punktzahl ermittelt werden. Die Gesamtpunktzahl nach Beantwortung des IPCAF ist ein Indikator für den erreichten Zustand bzw. Fortschritt im Prozess der Verbesserung. Die Ergebnisse können unter anderem genutzt werden, um einen konkreten Maßnahmenplan zu entwickeln (u.a. mit Hilfe der Anleitung zur Implementierung der WHO-Guidelines[2]) und Entscheidungsträger zu motivieren, hygienerelevante Strukturen und Aktivitäten zu intensivieren. Durch die regelmäßige Nutzung des IPCAF können Krankenhäuser/Gesundheitseinrichtungen ihre Fortschritte im zeitlichen Verlauf messen und auswerten.

Die WHO schlägt fünf Schritte zur effektiven Implementierung von Hygiene-/IPC-Programmen in Krankenhäusern/Gesundheitsreinrichtungen vor:

1. Bereiten Sie sich auf das Vorhaben vor.
2. **Führen Sie eine Ausgangsbewertung durch.**
3. Entwickeln und führen Sie einen Maßnahmenplan aus.
4. **Bewerten Sie die Auswirkungen.**
5. Setzen Sie das Programm langfristig fort.

Das IPCAF ist ein Instrument, welches insbesondere die Schritte 2 und 4 des beschriebenen Prozesses unterstützt. Schritt 2 „Führen Sie eine Ausgangsbewertung durch“ befasst sich mit der Beurteilung der aktuell vorliegenden Situation, einschließlich der Stärken und Schwächen, um eine Orientierung für den Maßnahmenplan zu schaffen. Schritt 4 „Bewerten Sie die Auswirkungen“ befasst sich mit der Beurteilung der Effektivität der im Rahmen des Maßnahmenplans durchgeführten Aktivitäten.

Wer sollte das IPCAF ausfüllen und nutzen?

- Fachkräfte des Gesundheitswesens, die für die Organisation und Umsetzung von Hygiene-/IPC-Aktivitäten verantwortlich sind und die ein fundiertes Wissen über die Hygiene-/IPC-Aktivitäten in Ihrem Krankenhaus/Ihrer Einrichtung aufweisen.
- Wenn es keine für Hygiene/IPC zuständigen Fachkräfte gibt oder noch kein Hygiene-/IPC-Programm eingerichtet wurde, kann das IPCAF vom leitenden Management (z.B. Klinikdirektor) genutzt werden.
- Das IPCAF bewertet das Krankenhaus/die Einrichtung als Ganzes. Bitte beachten Sie: in vielen Fällen wird für eine Formulierung verwendet, die mit einer direkten Ansprache verbunden ist („Sie“). Damit werden jedoch nicht einzelne Personen, sondern i.d.R. das Krankenhaus/die Einrichtung als Ganzes bezeichnet. Die Personen, die das IPCAF ausfüllen, sollten daher bei Bedarf Mitarbeiter anderer Bereiche (z.B. Arbeitsschutz und Arbeitsmedizin, Surveillance und Epidemiologie, Reinigung und Haustechnik, Umweltmedizin, Verwaltung etc.) konsultieren, um alle Fragen korrekt beantworten zu können.
- Das IPCAF wurde für den weltweiten Einsatz in Gesundheitseinrichtungen unabhängig von Größe, medizinischem Schwerpunkt und Modernisierungsgrad entwickelt.
- Bei einer Fremdbeurteilung von Einrichtungen durch externe Gutachter mit Hilfe des IPCAF sollten die Gutachter ebenfalls Hygiene/IPC-Fachleute sein, die die entsprechenden WHO-Leitlinien[1] kennen und verstehen.

Wie ist das IPCAF aufgebaut?

Das IPCAF ist entsprechend den Empfehlungen der WHO-Leitlinien[1] aufgebaut. Der Fragebogen gliedert sich in acht Abschnitte mit insgesamt 81 Indikatoren. Die Abschnitte entsprechen den acht IPC-Kernkomponenten der WHO-Leitlinien. Die 81 Indikatoren sind evidenzbasiert bzw. durch Expertenkonsens begründet und wurden als Fragen mit definierten Antworten umgesetzt, um eine bestmögliche Orientierungshilfe bei der Erhebung zu geben. Basierend auf der Gesamtbewertung über

alle acht Kernkomponenten erreicht die bewertete Einrichtung eine von vier Bewertungskategorien in Bezug auf die vorhandenen Hygienestrukturen und IPC-Aktivitäten:

1. **Unzureichend**: Die Hygiene-/IPC-Kernkomponenten-Implementierung ist mangelhaft. Eine deutliche Verbesserung ist erforderlich.
2. **Grundlegend**: Einige Aspekte der Hygiene-/IPC-Kernkomponenten sind umgesetzt, jedoch nicht in ausreichendem Umfang implementiert. Weitere Verbesserungen sind erforderlich.
3. **Intermediär**: Die meisten Hygiene-/IPC-Kernkomponenten sind angemessen implementiert. Das Krankenhaus/die Einrichtung sollte den Umfang und die Qualität der Umsetzung weiter verbessern und sich als nächsten Schritt auf die Entwicklung langfristiger Pläne zur Erhaltung und Weiterentwicklung bestehender Hygiene-/IPC-Programme und Aktivitäten konzentrieren.
4. **Fortgeschritten**: Die Hygiene-/IPC-Kernkomponenten sind nach den WHO-Empfehlungen und den Bedürfnissen des Krankenhauses/der Einrichtung vollständig umgesetzt.

<u>Wie funktioniert das IPCAF?</u>

Bitte wählen Sie bei der Beantwortung der Fragen jeweils die Antwort(en) aus, die die Situation in Ihrem Krankenhaus/Ihrer Einrichtung am Genausten beschreibt. Falls Ihnen Begrifflichkeiten einzelner Fragen oder die verwendete Terminologie nicht bekannt sind, wird empfohlen, sich mit den WHO-Guidelines[1] bzw. mit den in den jeweiligen Fußnoten aufgeführten Quellen vertraut zu machen. Mögliche Schwierigkeiten bei der Beantwortung der Fragen können ein Hinweis sein, dass bestimmte Hygienestrukturen oder IPC-Aktivitäten in Ihrem Krankenhaus/Ihrer Einrichtung noch nicht ausreichend entwickelt sind, und ermutigen zur Selbstreflektion und Verbesserung.

Bitte wählen Sie in der Regel nur eine Antwort pro Frage aus (Diese Fragen sind mit *„Wählen Sie bitte nur eine Antwort aus.“* gekennzeichnet oder einfache Ja/Nein-Fragen). Bei einigen Fragen sind auch Mehrfachantworten möglich (Diese Fragen sind mit dem Hinweis *„Bitte alle zutreffenden Antworten auswählen.“* gekennzeichnet).

Je nach Relevanz im jeweiligen Kontext werden den einzelnen Fragen bestimmte Punktzahlen zugeordnet. In jedem Abschnitt (Kernkomponente) kann eine maximale Punktzahl von 100 Punkten erreicht werden. Nachdem alle Fragen eines Abschnitts (Kernkomponente) beantwortet sind, werden die Punkte der Einzelfragen des Abschnitts summiert, so dass die Punktzahl je Abschnitt (Kernkomponente) berechnet werden kann. Die Gesamtpunktzahl wird durch Addition aller Punktzahlen der acht Abschnitte (Kernkomponenten) des Fragebogens berechnet.

<u>Ist das IPCAF geeignet für den Krankenhaus- oder Einrichtungsvergleich?</u>

Das primäre Ziel des IPCAF ist es, orientierend die aktuell vorliegende Situation der Hygiene und Infektionsprävention in der eigenen Einrichtung zu beurteilen und die Entwicklung und Verbesserung der Hygiene-/IPC-Aktivitäten im weiteren Verlauf durch wiederholte Anwendung des IPCAF zu überwachen. Die Nutzung des IPCAF ist <u>nicht</u> primär für den externen Vergleich oder ein Benchmarking gedacht. Der Vergleich verschiedener Gesundheitseinrichtungen kann bei Anwendung von korrekten und konsistenten Methoden möglich sein, sollte dann aber nur mit Vorsicht, vor allem bei Unterschieden hinsichtlich Größe, medizinischem Schwerpunkt und sozioökonomischen Rahmenbedingungen der teilnehmenden Einrichtungen durchgeführt werden.

Kernkomponente 1: Infection Prevention and Control (IPC) – Programm[3]

Frage	Antwort	Punkt-zahl
**1. Haben Sie ein Hygiene-/IPC-Programm?[3]** *Wählen Sie bitte nur eine Antwort aus*	Nein	0
	Ja, ohne klar definierte Ziele	5
	Ja, mit klar definierten Zielen und einem Jahresarbeitsplan	10
**2. Wird das Hygiene-/IPC-Programm durch ein Hygiene-/IPC-Team bestehend aus Hygiene-/IPC-Fachleuten[4] unterstützt?** *Wählen Sie bitte nur eine Antwort aus*	Nein	0
	Kein Team, nur ein Hygiene-/IPC-Hauptverantwortlicher/Ansprechpartner steht zur Verfügung	5
	Ja	10
**3. Weist das Hygiene-/IPC-Team mindestens eine Hygiene-/IPC-Vollzeitkraft (z.B. Krankenhaushygieniker/-in, Hygienefachkraft) oder Äquivalent (Pflegekraft oder Arzt/Ärztin zu 100% in Hygiene/IPC arbeitend) auf?** *Wählen Sie bitte nur eine Antwort aus*	Nein, keine Hygiene-/IPC-Fachkraft steht zur Verfügung	0
	Nein, nur eine Hygiene-/IPC-Teilzeitkraft vorhanden	2.5
	Ja, eine Hygiene/IPC-Fachkraft pro > 250 Betten vorhanden	5
	Ja, eine Hygiene/IPC-Fachkraft pro ≤250 Betten vorhanden	10
**4. Hat das Hygiene-/IPC-Team bzw. der Hygiene-/IPC-Hauptverantwortliche/Ansprechpartner ausreichend Zeit für die Hygiene-/IPC-Aufgaben?**	Nein	0
	Ja	10
**5. Besteht das Hygiene-/IPC-Team aus Pflegekräften und Ärzten?**	Nein	0
	Ja	10
**6. Haben Sie eine Hygiene-/IPC-Kommission[5] oder Ähnliches (z.B. Hygienekommission), welche das Hygiene-/IPC-Team unterstützt?**	Nein	0
	Ja	10
**7. Sind folgende Berufsgruppen in Ihrer Hygiene-/IPC-Kommission (oder einem Äquivalent) vertreten?**		
- Krankenhaus-/Einrichtungsleitung (z.B. Verwaltungsdirektion/Geschäftsführung, medizinisches Direktorat)	Nein	0
	Ja	5
- Leitendes klinisches Personal (z.B. Ärzteschaft, Pflegekräfte)	Nein	0
	Ja	2.5
- Facility Management (z.B. Bio-Sicherheit, Abfallwirtschaft, Beauftragte für Wasser, sanitäre Einrichtungen und Hygiene [WASH])	Nein	0
	Ja	2.5
**8. Haben Sie klar definierte Hygiene-/IPC-Zielsetzungen (z.B. in bestimmten kritischen Bereichen)?** *Wählen Sie bitte nur eine Antwort aus*	Nein	0
	Ja, nur Hygiene-/IPC-Zielsetzungen	2.5
	Ja, Hygiene-/IPC-Zielsetzungen (d.h. angemessene Maßnahmen zur Verbesserung) und messbare Ergebnisindikatoren	5

[3] Hygiene-/IPC-Programme sollten klar definierte Zielsetzungen basierend auf der lokalen Epidemiologie und Prioritäten gemäß einer Risikobewertung beinhalten sowie definierte Aufgaben und Aktivitäten, die sich mit der Prävention von nosokomialen Infektionen und antimikrobieller Resistenzen im Gesundheitswesen auseinandersetzen bzw. dazu beitragen. Es beinhaltet engagierte, ausgebildete Hygiene-/IPC-Fachleute. Für weitere Informationen siehe die *WHO Guidelines on core components of IPC programmes at the national and acute health care facility level* (http://www.who.int/infection-prevention/publications/core-components/en/, Zugriff am 06. August 2018).
[4] Hygiene-/IPC-Fachleute: Ärztliches oder pflegerisches Personal, ausgebildet in einer zertifizierten Hygiene-/IPC-Ausbildung
[5] Ein Hygiene-/IPC-Team besteht aus dafür angestellten Hygiene-/IPC-Fachleuten. Eine Hygiene-/IPC-Kommission ist eine multidisziplinäre Gruppe mit Interessensvertretern des Krankenhauses/der Einrichtung, die mit dem Hygiene-/IPC-Team interagieren und dieses beraten.

	Ja, Hygiene-/IPC-Zielsetzungen, messbare Ergebnisindikatoren und klare Ziele für die Zukunft	10
**9. Unterstützt Sie die Krankenhaus-/Einrichtungsleitung bei dem Hygiene-/IPC-Programm durch:**		
- ein zugewiesenes Budget für das Hygiene-/IPC-Programm (für Hygiene-/IPC-Aktivitäten, auch inkl. Gehältern)?	Nein	0
	Ja	5
- eine nachweisliche Unterstützung für die Hygiene-/IPC-Ziele und Indikatoren innerhalb des Krankenhauses/der Einrichtung (z.B. bei Vorstandsmeetings, Vorstandsrunden, Teilnahme an Morbiditäts- und Mortalitätssitzungen)?	Nein	0
	Ja	5
**10. Erhält Ihr Krankenhaus/Ihre Einrichtung in der täglichen Routine Unterstützung durch ein mikrobiologisches Labor?** *Wählen Sie bitte nur eine Antwort aus*	Nein	0
	Ja, aber Ergebnisse werden nicht verlässlich (d.h. zeitnah und qualitativ ausreichend) übermittelt	5
	Ja, und Ergebnisse werden verlässlich (d.h. zeitnah und qualitativ ausreichend) übermittelt	10
**Zwischensumme**		**/100**

Kernkomponente 2: Infection Prevention and Control (IPC) – Leitlinien

Frage	Antwort	Punkt-zahl
**1. Hat Ihr Krankenhaus/Ihre Einrichtung die Expertise für die Entwicklung oder Anpassung von Hygiene-Leitlinien?**	Nein	0
	Ja	7.5
**2. Hat Ihr Krankenhaus/Ihre Einrichtung Hygiene-/IPC-Leitlinien für:**		
- Standardbarrieremaßnahmen?	Nein	0
	Ja	2.5
- Händehygiene?	Nein	0
	Ja	2.5
- Übertragungsweg-bezogene Barrieremaßnahmen[6]?	Nein	0
	Ja	2.5
- Ausbruchsmanagement?	Nein	0
	Ja	2.5
- Prävention von postoperativen Wundinfektionen[7]?	Nein	0
	Ja	2.5
- Prävention von Katheter-assoziierten Blutstrominfektionen?	Nein	0
	Ja	2.5
- Prävention von nosokomialen Pneumonien (sowohl Beatmungs- als auch nicht-Beatmungs-assoziiert)?	Nein	0
	Ja	2.5
- Prävention von Katheter-assoziierten Harnweginfektionen?	Nein	0
	Ja	2.5
- Prävention von Übertragungen von multiresistenten Erregern (MRE)?	Nein	0
	Ja	2.5
- Desinfektion und Sterilisation?	Nein	0
	Ja	2.5
- Gesundheit- und Arbeitsschutz des Personals[8]?	Nein	0
	Ja	2.5
- Injektionssicherheit (z.B. Sicherheitsnadeln)?	Nein	0
	Ja	2.5
- Abfallwirtschaft?	Nein	0
	Ja	2.5
- Antibiotic Stewardship[9]?	Nein	0
	Ja	2.5
**3. Sind die Hygiene-/IPC-Leitlinien in Ihrem Krankenhaus/Ihrer Einrichtung konsistent mit nationalen/internationalen Leitlinien (sofern diese existieren)?**	Nein	0
	Ja	10
	Nein	0

[6] Übertragungsweg-bezogene Barrieremaßnahmen werden zusätzlich zu Standard-Barrieremaßnahmen bei solchen Patienten angewendet, die mit bestimmten infektiösen Erregern infiziert oder kolonisiert sein könnten und für die zusätzliche Barrieremaßnahmen zur Vermeidung von Übertragungen notwendig sind. Die Übertragungsweg-bezogenen Barriere-Maßnahmen Sie sind spezifisch für die basieren auf den jeweiligen Übertragungswegen der Erreger und deren Übertragungsweg (z.B. Kontaktübertragung vs. Tröpfchenübertragung). Weiterführende Informationen finden Sie in den *Guidelines for Isolation Precautions* der United States Centers for Disease Control and Prevention (https://www.cdc.gov/infectioncontrol/pdf/guidelines/isolation-guidelines.pdf) (Zugriff am 06. August 2018).

[7] Falls keine chirurgischen Eingriffe an Ihrem Krankenhaus/Ihrer Einrichtung stattfinden, wählen Sie bitte „Ja“ aus.

[8] Enthält Aspekte der Verbesserung der Arbeitsbedingungen, Erkennen von berufsbedingten Erkrankungen, Gesundheitsmonitoring von Beschäftigten, Einstellungsuntersuchungen und Impfungen.

[9] „Antibiotic Stewardship“ bezeichnet Bemühungen durch einen angemessenen und rationalen Einsatz von Antibiotika das Outcome der Patienten zu verbessern und gleichzeitig Resistenzentwicklung und -verbreitung zu reduzieren. Weiterführende Informationen finden Sie im *WHO Global Framework for Development & Stewardship to Combat Antimicrobial Resistance* (http://www.who.int/antimicrobial-resistance/global-action-plan/UpdatedRoadmap-Global-Framework-for-Development-Stewardship-to-combatAMR_2017_11_03.pdf , Zugriff am 06. August 2018).

**4. Erfolgt bei der Umsetzung der Hygiene-/IPC-Leitlinien eine Anpassung an den lokalen Bedarf und an die lokalen Ressourcen unter Wahrung zentraler Hygiene-/IPC-Standards[10]?**	Ja	10
**5. Ist klinisches Personal zusätzlich zum Hygiene-/IPC-Personal an der Planung und Durchführung der Umsetzung der Hygiene-/IPC-Leitlinien beteiligt?**	Nein	0
	Ja	10
**6. Sind relevante Interessensgruppen (z.B. medizinische Leitung und leitende Pflegekräfte, Krankenhaus-/Einrichtungsmanagement, Qualitätsmanagement) zusätzlich zum Hygiene-/IPC-Personal an der Entwicklung und Anpassung der Hygiene-/IPC-Leitlinien beteiligt?**	Nein	0
	Ja	7.5
**7. Wird das medizinische Personal spezifisch zu den Inhalten neuer oder überarbeiteter Hygiene-/IPC-Leitlinien geschult, sobald diese veröffentlicht werden?**	Nein	0
	Ja	10
**8. Werden in Ihrem Krankenhaus/Ihrer Einrichtung die Umsetzung mindestens einiger der Hygiene-/IPC-Leitlinien regelmäßig kontrolliert?**	Nein	0
	Ja	10
**Zwischensumme**		**/100**

[10] Das Hygiene-/IPC-Team überprüft sorgfältig Hygiene-/IPC-Leitlinien, um Aktivitäten nach Bedarf und Ressourcen - unter Wahrung zentraler Hygiene-/IPC-Standards - zu priorisieren.

Kernkomponente 3: Infection Prevention and Control (IPC) – Ausbildung und Schulung

Frage	Antwort	Punkt-zahl
**1. Gibt es Personal mit ausreichenden Kenntnissen (in Hygiene/IPC und/oder Infektiologie), um eine Hygiene-/IPC-Schulung zu leiten?**	Nein	0
	Ja	10
**2. Gibt es zusätzliche Nicht-Hygiene-/IPC-Team-Mitarbeiter (z.B. hygienebeauftragte Pflegekräfte oder Ärzte, „Champions“) mit ausreichenden Kenntnissen, um als Trainer oder Mentoren zu fungieren?** *Wählen Sie bitte nur eine Antwort aus*	Nein	0
	Ja	10
**3. Wie häufig wird das klinische Personal in Ihrem Krankenhaus/Ihrer Einrichtung in Bezug auf Hygiene-/IPC geschult?** *Wählen Sie bitte nur eine Antwort aus*	Selten bis nie	0
	Nur bei der Einweisung neuer Mitarbeiter	5
	Bei der Einweisung neuer Mitarbeiter und als regelmäßige Hygiene-/IPC-Schulung für alle klinisch tätigen Mitarbeiter (mindestens einmal jährlich), allerdings nicht verpflichtend	10
	Bei der Einweisung neuer Mitarbeiter und regelmäßige, verpflichtende Hygiene-/IPC-Schulung für alle klinisch tätigen Mitarbeiter (mindestens einmal jährlich)	15
**4. Wie häufig erhalten andere Mitarbeiter, die direkt in die Patientenversorgung eingebunden sind (z.B. Reinigungs- und Hilfs-Servicepersonal), Hygiene-/IPC-Schulungen?** *Wählen Sie bitte nur eine Antwort aus*	Selten bis nie	0
	Nur bei der Einweisung neuer Mitarbeiter	5
	Bei der Einweisung neuer Mitarbeiter und als regelmäßige Hygiene-/IPC-Schulung für entsprechendes Personal (mindestens einmal jährlich), aber nicht verpflichtend	10
	Bei der Einweisung neuer Mitarbeiter und als regelmäßige, verpflichtende Hygiene-/IPC-Schulung für entsprechendes Personal (mindestens einmal jährlich)	15
**5. Erhalten Mitarbeiter der Verwaltung und des Managements eine Basis-Hygiene-/IPC-Schulung?**	Nein	0
	Ja	5
**6. Wie wird das Personal geschult?** *Wählen Sie bitte nur eine Antwort aus*	Es gibt keine Schulungsveranstaltungen	0
	Nur durch schriftliche Informationen und/oder nur durch mündliche Unterweisung und/oder nur E-Learning	5
	Zusätzlich durch interaktive Trainingseinheiten (z.B. Simulationstraining und/oder Schulung am Krankenbett)	10
**7. Gibt es regelmäßige Auswertungen der Wirksamkeit von Schulungsprogrammen (z.B. Händehygiene-Audits, andere Wissensüberprüfungen)?** *Wählen Sie bitte nur eine Antwort aus*	Nein	0
	Ja, aber nicht regelmäßig	5
	Ja, regelmäßig (mindestens einmal jährlich)	10
**7. Sind Hygiene-/IPC-Schulungen in die klinische Praxis und in die Ausbildung anderer Fachgebiete integriert (z.B. beinhaltet die Ausbildung von Chirurgen Aspekte von Hygiene/IPC)?** *Wählen Sie bitte nur eine Antwort aus*	Nein	0
	Ja, in einigen Fachdisziplinen	5
	Ja, in allen Fachdisziplinen	10

**9. Gibt es speziell angepasste Hygiene-/IPC-Schulungen für Patienten oder Familienmitglieder, um das Potential von nosokomialen Infektionen zu minimieren (z.B. für immunsupprimierte Patienten, Patienten mit invasiven Zugängen/Devices, Patienten mit MRE)?**	Nein	0
	Ja	5
**10. Wird eine Weiterbildung für Hygiene-/IPC-Mitarbeiter sichergestellt (z.B. durch regelmäßige Teilnahme an Konferenzen, Kongressen, Kursen)?**	Nein	0
	Ja	10
**Zwischensumme**		**/100**

Kernkomponente 4: Surveillance von nosokomialen Infektionen

Frage	Antwort	Punkt-zahl
**Organisation von Surveillance**		
**1. Ist Surveillance eine klar definierte Komponente Ihres Hygiene-/IPC-Programms?**	Nein	0
	Ja	5
**2. Haben Sie Personal, das für Surveillance-Tätigkeiten verantwortlich ist?**	Nein	0
	Ja	5
**3. Ist das für Surveillance verantwortliche Personal in Grundlagen der Epidemiologie, der Surveillance und der Hygiene/IPC geschult (d.h. Kenntnisse über Surveillance-Methoden sowie über Datenmanagement und –interpretation)?**	Nein	0
	Ja	5
**4. Haben Sie IT-Unterstützung um Ihre Surveillance durchzuführen (z.B. IT-Ausrüstung, mobile Technologien, elektronische Patientenakten)?**	Nein	0
	Ja	5
**Prioritäten für Surveillance – definiert nach dem Versorgungsschwerpunkt**		
**5. Findet eine Priorisierung statt, um nosokomiale Infektionen zu bestimmen, die für die Surveillance im lokalen Zusammenhang bedeutend sind (d.h. die Identifizierung von Infektionen, die wesentlich zu Morbidität und Mortalität im Krankenhaus/in der Einrichtung beitragen)?[11]**	Nein	0
	Ja	5
**6. Führen Sie Surveillance durch für:**		
- Postoperative Wundinfektionen?[12]	Nein	0
	Ja	2.5
- Device-assoziierte Infektionen (z.B. Katheter-assoziierte Harnweginfektionen, zentral-venöser Katheter (ZVK)-assoziierte Blutstrominfektionen, peripher-venöser Katheter (PVK)-assoziierte Blutstrominfektionen, Beatmungs-assoziierte Pneumonien)?	Nein	0
	Ja	2.5
- Klinisch-definierte Infektionen (anhand klinischer Symptome, z.B. bei Nicht-Verfügbarkeit bzw. Nicht-Vorhandenheit von mikrobiologischen Untersuchungen)?	Nein	0
	Ja	2.5
- Kolonisation oder Infektion durch MRE[13] gemäß der lokalen epidemiologischen Situation	Nein	0
	Ja	2.5
- Infektionen mit Epidemiegefahr entsprechend lokaler Prioritäten (z.B. Norovirus, Influenza, Tuberkulose (TB), schweres akutes respiratorisches Syndrom (SARS), Ebola, Lassa-Fieber)?	Nein	0
	Ja	2.5
- Infektionen bei vulnerablen Patienten (z.B. Neugeborene, Intensivpatienten, immunsupprimierte Patienten, Verbrennungspatienten)?[14]	Nein	0
	Ja	2.5
- Infektionen, die Mitarbeiter in klinischen, laboratorischen oder anderen Bereichen betreffen können (z.B. Hepatitis B oder C, HIV, Influenza)?	Nein	0
	Ja	2.5
**7. Evaluieren Sie regelmäßig, ob Ihre Surveillance an die aktuellen Bedürfnissen und Prioritäten angepasst ist?[11]**	Nein	0
	Ja	5
**Methoden der Surveillance**		

[11] Ein Priorisierungsprozess sollte durchgeführt werden, um zu bestimmen, welche nosokomiale Infektionen entsprechend dem lokalen Kontext unter Surveillance genommen werden sollten (z.B. besonders gefährdete Bereiche oder Patienten). Dabei werden entsprechende Quellen genutzt (siehe *Interim practical manual* der WHO zur Implementierung der WHO-Guidelines zur Infektionsprävention und –kontrolle unter http://www.who.int/infection-prevention/tools/core-components/en/, Zugriff am 06. August 2018)
[12] Falls keine chirurgischen Eingriffe an Ihrem Krankenhaus/Ihrer Einrichtung stattfinden, wählen Sie bitte „Ja" aus.
[13] MRE (multiresistente Erreger): Nicht-Ansprechen auf mindestens ein Antibiotikum aus drei oder mehr Antibiotikaklassen.
[14] Sofern keine vulnerablen Patientengruppen in Ihrem Krankenhaus/Ihrer Einrichtung behandelt werden, wählen Sie bitte „Ja" aus.

**8. Verwenden Sie zuverlässige Surveillance-Falldefinitionen (definierte Zähler und Nenner nach internationalen Definitionen (z.B. CDC NHSN/ECDC)[15] oder falls angepasste Definitionen verwendet werden, erfolgt eine Anpassung durch einen sorgfältigen evidenzbasierten Anpassungsprozess und eine Expertenkonsultation)?**	Nein	0
	Ja	5
**9. Verwenden Sie standardisierte Datenerfassungsmethoden (z.B. aktive prospektive Surveillance) nach internationalen Surveillance-Protokollen (z.B. CDC NHSN/ECDC) oder falls angepasste Definitionen verwendet werden, erfolgt eine Anpassung durch einen sorgfältigen evidenzbasierten Anpassungsprozess und eine Expertenkonsultation?**	Nein	0
	Ja	5
**10. Führen Sie eine regelmäßige Überprüfung der Datenqualität durch (z.B. Bewertung von Fallberichten, Überprüfung von mikrobiologischen Ergebnissen, Bestimmung geeigneter Nennerdaten usw.)?**	Nein	0
	Ja	5
**11. Haben Sie eine ausreichende Mikrobiologie- und Laborkapazität zur Unterstützung der Surveillance?**<br>*Wählen Sie bitte nur eine Antwort aus*	Nein	0
	Ja, eine Unterscheidung zwischen Gram-positiven und Gram-negativen Erregern ist möglich, der Erreger kann aber nicht genauer identifiziert werden	2.5
	Ja, es erfolgt eine verlässliche und zeitnahe Identifizierung des Erregers	5
	Ja, es erfolgt eine verlässliche und zeitnahe Identifizierung des Erregers und der Antibiotikaresistenzen (Antibiogramm)	10
**Informationsanalyse und -verbreitung/Datenverwendung, Datenverknüpfung und -verwaltung**		
**12. Werden die Surveillance-Daten für die Verbesserung der Hygiene-/IPC-Praxis in Ihrem Krankenhaus/Ihrer Einrichtung bzw. auf einzelnen Stationen verwendet?**	Nein	0
	Ja	5
**13. Analysieren Sie antimikrobielle Resistenzen regelmäßig (z.B. vierteljährlich/halbjährlich/jährlich)?**	Nein	0
	Ja	5
**14. Geben Sie regelmäßig (z.B. vierteljährlich/halbjährlich/jährlich) Feedback über aktuelle Surveillance-Daten an:**		
- Klinisches Personal (Ärzte/Pflegepersonal)?	Nein	0
	Ja	2.5
- Klinik-/Abteilungsleitung?	Nein	0
	Ja	2.5
- Die Hygiene-/IPC-Kommission?	Nein	0
	Ja	2.5
- Nicht-klinisches Management/Verwaltung (Geschäftsführer/Finanzvorstand)	Nein	0
	Ja	2.5

[15] United States Centers for Disease Control and Prevention (CDC) National Healthcare Safety Network (NHSN) (https://www.cdc.gov/nhsn/index.html, Zugriff am 06. August 2018); European Centre for Disease Prevention and Control (ECDC) (https://ecdc.europa.eu/en/about-us/partnerships-and-networks/disease-and-laboratory-networks/hai-net, Zugriff am 06. August 2018).

**15. In welcher Form geben Sie Feedback zu aktuellen Surveillance-Daten?**<br>**(mindestens jährlich)**<br>*Wählen Sie bitte nur eine Antwort aus*	Kein Feedback	0
	Nur durch schriftliche/mündliche Information	2.5
	Durch Präsentation und interaktive problemorientierte Lösungsfindung	7.5
**Zwischensumme**		**/100**

Kernkomponente 5: Multimodale Strategien[16] zur Implementierung von Infection Prevention and Control (IPC) – Interventionen

Frage	Antwort	Punkt-zahl
**1. Verwenden Sie multimodale Strategien[16] zur Implementierung von Hygiene-/IPC-Interventionen?**	Nein	0
	Ja	15
**2. Beinhalten Ihre multimodalen Strategien einige oder alle der folgenden Elemente (a bis e)?** *Bitte wählen Sie die zutreffendste Antwort pro Element*	**a) Veränderungen im System / der Infrastruktur**	
	Dieses Element ist nicht Inhalt der multimodalen Strategie.	0
	Interventionen zur Sicherstellung der notwendigen Infrastruktur und eine ständige Verfügbarkeit der entsprechenden Versorgungsgüter sind vorhanden	5
	Es existieren Interventionen, um die notwendige Infrastruktur sowie die ständige Verfügbarkeit von Versorgungsgütern zu gewährleisten. Die Interventionen berücksichtigen Fragen der Ergonomie[17] und Zugänglichkeit (z.B. was ist der beste Platz für die Lagerung eines ZVK-Sets und des zugehörigen Materials)	10
	**b) Schulung und Training**	
	Dieses Element ist nicht Inhalt der multimodalen Strategie.	0
	Schulung erfolgt durch schriftliche Informationen und/oder nur durch mündliche Unterweisung und/oder nur durch E-Learning	5
	Zusätzliche interaktive Trainingseinheiten (z.B. Simulationstraining und/oder Schulung am Krankenbett) sind vorhanden	10
	**c) Monitoring (Überwachung) und Feedback**	
	Dieses Element ist nicht Inhalt der multimodalen Strategie.	0
	Monitoring der Compliance von Prozess- oder Ergebnisindikatoren (z.B. Audits zur Händehygiene oder zum Umgang mit Gefäßkathetern) erfolgt	5
	Überwachung der Compliance und Erteilung eines zeitnahen Feedbacks der Ergebnisse an das klinische Personal sowie andere wichtige Mitarbeiter erfolgen	10
	**d) Kommunikation und Erinnerungssysteme**	
	Dieses Element ist nicht Inhalt der multimodalen Strategie.	0
	Erinnerungssystemen, Plakaten oder anderen Sensibilisierungswerkzeugen zur Förderung der Intervention werden verwendet	5

[16] Siehe Definition unter http://www.who.int/infection-prevention/publications/ipc-cc-mis.pdf?ua=1, Zugriff am 06. August 2018. Die Verwendung von multimodalen Strategien kann als Evidenz-basierter Ansatz hilfreich sein, nachhaltige Änderungen des Systems und des Verhaltens zur Implementierung von Hygiene-/IPC-Interventionen zu etablieren. Multimodale Strategien: ≥3 Komponenten, die in einer integrierten Weise eingebracht werden, um eine Verbesserung des Ergebnisses zu erzielen und das Verhalten zu ändern (z.B. Händehygiene). Die Komponenten können beinhalten: (i) Systemwechsel (z.B. die notwendige Infrastruktur, die Versorgung und die Humanressourcen zur Verfügung zu stellen), (ii) Aus- und Weiterbildung der Mitarbeiter im Gesundheitswesen und der Schlüsselpersonen (z.B. Führungskräfte), (iii) Überwachung von Infrastrukturen, Praktiken, Prozessen, Ergebnissen und Bereitstellung von Datenfeedback, (iv) Erinnerungssysteme am Arbeitsplatz und angemessene Kommunikation, (v) Kulturveränderung im Rahmen der Etablierung oder Stärkung eines Sicherheitsklimas. Dazu gehören auch Instrumente, wie Checklisten und Bundles, die von multidisziplinären Teams unter Berücksichtigung der lokalen Gegebenheiten entwickelt wurden. Alle fünf Komponenten sollten in Anpassung an die lokalen Umstände und unter regelmäßiger Bewertung berücksichtigt werden. Wenn notwendig sollten Veränderungen eingeleitet werden. Erkenntnisse aus den Implementierungswissenschaften legen nahe, dass Interventionen, die lediglich eine Komponente der multimodalen Strategien berücksichtigen, zu weniger nachhaltigen Ergebnissen führten. Weitere Informationen unter http://www.who.int/infection-prevention/publications/ipc-cc-mis.pdf?ua=1, Zugriff am 06. August 2018, sowie dem *Interim practical manual* der WHO zur Implementierung der WHO-Guidelines zur Infektionsprävention und –kontrolle unter http://www.who.int/infection-prevention/tools/core-components/en/, Zugriff am 06. August 2018.

[17] Ergonomie: menschliche Faktoren oder ein Verständnis von Interaktionen zwischen Menschen und Elementen eines Systems zur Optimierung des menschlichen Wohlbefindens und zur Erhöhung der Gesamtsystemleistung und zur Vermeidung von menschlichen Fehlern. Weitere Informationen unter: http://www.health.org.uk/sites/health/files/IntegratingHumanFactorsWithInfectionAndPreventionControl.pdf, Zugriff am 06. August 2018.

	Zusätzliche Methoden/Initiativen zur besseren Kommunikation innerhalb der Teams (ggf. auch Stations- und Fachdisziplin-übergreifend), z.B. durch die Einrichtung regelmäßiger Fallkonferenzen und Feedback-Runden kommen zur Anwendung	10
	**e) Sicherheitsklima und Kulturwandel**	
	Dieses Element ist nicht Inhalt der multimodalen Strategie.	0
	Die Leitung/das Management zeigt eine sichtbare Unterstützung und fördert als Vorbild und Champion einen adaptiven Ansatz[18] sowie eine Kultur, die Hygiene/IPC, Patientensicherheit und die Qualitätssicherung unterstützt.	5
	Zusätzlich werden Teams und einzelne Mitarbeiter gestärkt, sodass diese sich als Gestalter der Intervention begreifen (z.B. durch partizipative Feedback-Runden)	10
**3. Ist ein interdisziplinäres Team beteiligt, um multimodale Hygiene-/IPC-Strategien umzusetzen?**	Nein	0
	Ja	15
**4. Stimmen Sie sich regelmäßig mit Kollegen aus dem Qualitätsmanagement und der Patientensicherheit ab, um multimodale Hygiene-/IPC-Strategien zu entwickeln und zu fördern?**	Nein	0
	Ja	10
**5. Beinhalten Ihre Strategien Bundles[19] oder Checklisten?**	Nein	0
	Ja	10
**Zwischensumme**		**/100**

[18] Adaptive Ansätze betrachten die Verhaltens-, Organisations- und Kulturkomplexität im Gesundheitswesen. Sie zielen darauf ab, ein lokales Sicherheitsklima zu etablieren und lokale Teams zu motivieren bestmöglich zu handeln, indem die Einstellung, die Denkweise sowie die Werte des klinischen Personals beeinflusst werden. Das beinhaltet zum Beispiel Führungspersonen miteinzubeziehen, Zusammenarbeit und Teamwork zu verbessern und den einzelnen Mitarbeitern Einfluss auf die Intervention zu ermöglichen. Mehr Informationen finden Sie unter https://www.ahrq.gov/professionals/education/curriculum-tools/cusptoolkit/index.html, Zugriff am 06. August 2018.

[19] Bundles: Bündel evidenzbasierter Praktiken, mit Fokus auf die Verbesserung der Behandlungsprozesse in einer strukturierten Weise, z.B. Verbesserung der Katheter-Anlage. Bundles sind nicht gleichbedeutend mit multimodalen Strategien, vielmehr werden Bundles zur Erleichterung der Implementierung von Hygiene-/IPC-Maßnahmen eingesetzt, idealerweise im Kontext von multimodalen Strategien. Multimodale Strategien sind ein weitaus umfassender Ansatz.

Kernkomponente 6: Monitoring/Audits der Hygiene-/IPC-Praxis und Feedback

Frage	Antwort	Punktzahl
**1. Haben Sie geschultes Personal, das für das Monitoring/die Auditierung von Hygiene-/IPC-Praktiken und für Feedback verantwortlich ist?**	Nein	0
	Ja	10
**2. Haben Sie einen gut definierten Monitoringplan mit klaren Zielvorstellungen und Aktivitäten (einschließlich Tools zur systematischen Erfassung von Daten)?**	Nein	0
	Ja	7.5
**3. Welche Prozesse und Indikatoren überwachen Sie in Ihrem Krankenhaus/Ihrer Einrichtung?** *Bitte alle zutreffenden Antworten auswählen*	Keine	0
	Händehygiene-Compliance (mit dem WHO Händehygiene-Beobachtungs-Tool[20] oder einem Äquivalent)	5
	Intravaskuläre Katheter-Anlage und/oder Pflege	5
	Wundverbandswechsel	5
	Barrieremaßnahmen und Isolierung, um die Ausbreitung von MRE zu verhindern	5
	Reinigung der Stationen	5
	Desinfektion und Sterilisation von medizinischen Geräten/Instrumenten	5
	Verbrauch/Verwendung von alkoholhaltigem Handdesinfektionsmittel oder Seife	5
	Verbrauch/Verwendung von Antibiotika	5
	Abfallwirtschaft	5
**4. Wie häufig führen Sie den WHO-Händehygiene Selbstbewertungs-Fragebogen (WHO-Hand Hygiene Self-Assessment Framework Survey)[21] in Ihrer Arbeitsroutine durch?** *Wählen Sie bitte nur eine Antwort aus*	Nie	0
	Periodisch, aber nicht regelmäßig	2.5
	Mindestens einmal jährlich	5
**5. Geben Sie Feedback zu Hygiene-/IPC-Audit-Berichten (z.B. Feedback zu Händehygiene-Compliancebeobachtungen oder anderen Prozessen)?** *Bitte alle zutreffenden Antworten auswählen*	Kein Feedback	0
	Ja, innerhalb des Hygiene-/IPC-Teams	2.5
	Ja, den Abteilungsleitern und den Führungskräften in den überprüften Bereichen	2.5
	Ja, dem klinisch tätigen Personal	2.5
	Ja, dem Hygiene-/IPC-Komitee oder dem Qualitätsmanagementausschuss oder einem Äquivalent	2.5
	Ja, dem Krankenhausmanagement und der Verwaltung	2.5
**6. Wird die Berichterstattung von Monitoring-Daten regelmäßig (mindestens einmal jährlich) durchgeführt?**	Nein	0
	Ja	10

[20] WHO Händehygiene-Monitoring und Feedback-Tools finden Sie unter http://www.who.int/gpsc/5may/tools/evaluation_feedback/en/, Zugriff am 06. August 2018.

[21] Das *WHO Hand Hygiene Self-Assessment Framework* finden Sie unter http://www.who.int/gpsc/country_work/hhsa_framework_October_2010.pdf?ua=1, Zugriff am 06. August 2018.

**7. Werden Monitoring und Feedback zu Hygiene-/IPC-Prozessen in einer „sanktionsfreien“, „vorwurfsfreien“ institutionellen Kultur durchgeführt, die auf Verbesserung und mögliche Verhaltensänderung ausgerichtet ist?**	Nein	0
	Ja	5
**8. Erheben und bewerten Sie Faktoren der Sicherheitskultur in Ihrem Krankenhaus/Ihrer Einrichtung (z.B. durch die Verwendung anderer Fragebögen wie HSOPSC, SAQ, PSCHO, HSC)[22]?**	Nein	0
	Ja	5
**Zwischensumme**		**/100**

[22] HSOPSC: Hospital survey on patient safety culture; SAQ: Safety attitudes questionaire; PSCHO: Patient safety climate in healthcare organizations; HSC: Hospital safety climate scale. Eine Zusammenfassung dieser Fragebögen finden Sie hier: Colla JB, et al. Measuring patient safety climate: a review of survey. Qual Saf Health Care. 2005;14(5):364-6 (https://www.ncbi.nlm.nih.gov/pubmed/16195571, Zugriff am 06. August 2018).

Kernkomponente 7: Arbeitsaufwand, Personal und Bettenbelegung[23]

Frage	Antwort	Punkt-zahl
**Personalbesetzung**		
**1. Legen Sie eine angemessene Personalausstattung in Hinblick auf den Arbeitsaufwand bei der Krankenversorgung entsprechend nationaler Standards oder mit einem Personalbedarfsanalyse-Tool (z.B. die Methode des *WHO Workload indicators of staffing need*[24]) fest?**	Nein	0
	Ja	5
**2. Halten Sie in Ihrem Krankenhaus/Ihrer Einrichtung einen definierten Personal-zu-Patienten-Schlüssel[25] (d.h. auf Basis der WHO oder nationaler Standards) aufrecht?** *Wählen Sie bitte nur eine Antwort aus*	Nein	0
	Ja, für das Personal in weniger als 50% der Stationen	5
	Ja, für das Personal in mehr als 50% der Stationen	10
	Ja, für das gesamte Personal des Krankenhauses/der Einrichtung	15
**3. Haben Sie ein System, um auf die Ergebnisse der Personalbedarfsbewertung zu reagieren, wenn die Personalbestände als unzureichend eingestuft werden?**	Nein	0
	Ja	10
**Bettenbelegung**		
**4. Entspricht das Design Ihrer Stationen internationalen Standards[26] in Bezug auf die Bettenkapazität?** *Wählen Sie bitte nur eine Antwort aus*	Nein	0
	Ja, aber nur in bestimmten Abteilungen	5
	Ja, in allen Abteilungen (einschließlich Notaufnahme und Kinderheilkunde)	15
**5. Wird in Ihrem Krankenhaus/Ihrer Einrichtung ein Bett mit jeweils nur einem Patienten belegt?** *Wählen Sie bitte nur eine Antwort aus*	Nein	0
	Ja, aber nur in bestimmten Abteilungen	5
	Ja, in allen Abteilungen (einschließlich Notaufnahme und Kinderheilkunde)	15
**6. Platzieren Sie Betten mit Patienten außerhalb von den Zimmern auf dem Flur („Flurbetten") (Frage schließt Betten in der Notaufnahme ein)?** *Wählen Sie bitte nur eine Antwort aus*	Ja, öfter als zweimal pro Woche	0
	Ja, seltener als zweimal pro Woche	5
	Nein	15
**7. Besteht in Ihrem Krankenhaus/Ihrer Einrichtung ein ausreichender Abstand von > 1 Meter zwischen den Patientenbetten?** *Wählen Sie bitte nur eine Antwort aus*	Nein	0
	ja, aber nur in bestimmten Abteilungen	5
	Ja, in allen Abteilungen (einschließlich Notaufnahme und Kinderheilkunde)	15
**8. Haben Sie ein System, um festzustellen und zu reagieren, wenn die adäquate Bettenkapazität überschritten ist?**	Nein	0
	Ja, das liegt in der Verantwortung der Abteilungsleitung (z.B. Chefärztin/Chefarzt)	5

[23] Zur Beantwortung insbesondere der Fragen der Kernkomponente 7 ist es hilfreich Kontakt zu den entsprechenden zuständigen Personen/Teams in Ihrem Krankenhaus/Ihrer Einrichtung aufzunehmen, um die Fragen korrekt beantworten zu können.

[24] Die Methode der WHO *Workload indicators of staffing need* (WHO-Arbeitsaufwand-Indikatoren zur Ermittlung des Personalbedarfs) bietet Managern im Gesundheitswesen einen systematischen Weg, um festzustellen, wie viele Mitarbeiter eines bestimmten Typs benötigt werden, um die Arbeitsbelastung einer bestimmten Gesundheitseinrichtung zu bewältigen und die Entscheidungsfindung dahingehend zu unterstützen (http://www.who.int/hrh/resources/wisn_user_manual/en/, Zugriff am 06. August 2018).

[25] Der Personal-zu-Patienten-Schlüssel berücksichtigt sämtliches Personal, welches in den Bereichen der Patientenverorgung und des Patientenservice arbeitet. Dies schließt klinisches Personal (Ärzteschaft, Pflege, medizinische Assistenten, etc.), Laborpersonal und andere Mitarbeiter (z.B. Reinigungspersonal) mit ein.

[26] Die *WHO Essential environmental health standards in health care guidance* (WHO-Umweltgesundheitsnormen im Gesundheitswesen) bieten Leitlinien für Standards, die für das Gesundheitswesen in Mittel- und Niedrig-Ressourcen-Ländern erforderlich sind. Die Richtlinien wurden für den Einsatz von Entscheidungsträgern im Gesundheitswesen sowie Architekten, Stadtplanern, Wasser- und Sanitätspersonal, ärztlichen und pflegerischen Mitarbeitern und anderen Gesundheitsdienstleistern sowie Gesundheitsförderern erstellt (http://www.who.int/water_sanitation_health/publications/ehs_hc/en/, Zugriff am 06. August 2018).

Wählen Sie bitte nur eine Antwort aus	Ja, das liegt in der Verantwortung der Krankenhausverwaltung/des Managements	10
**Zwischensumme**		**/100**

Kernkomponente 8: Strukturgestaltung, Materialien und Ausstattung für Hygiene-/IPC auf Krankenhaus-/Einrichtungsebene[27]

Frage	Antwort	Punkt-zahl
**Wasserversorgung**		
**1. Ist für alle Verwendungszwecke jederzeit eine Wasserversorgung in ausreichender Menge verfügbar (z.B. für Händewaschen, Trinken, Körperpflege und -hygiene, medizinische Tätigkeiten, Sterilisation, Dekontamination, Reinigung und Wäsche)?** *Wählen Sie bitte nur eine Antwort aus*	Nein, im Durchschnitt < 5 Tage pro Woche	0
	Ja, im Durchschnitt ≥ 5 Tage pro Woche oder jeden Tag, aber nicht in ausreichender Menge	2.5
	Ja, jeden Tag und in ausreichender Menge	7.5
**2. Ist eine zuverlässige Trinkwasserquelle vorhanden und auf jeder Station jederzeit für Mitarbeiter, Patienten und Angehörige zugänglich?** *Wählen Sie bitte nur eine Antwort aus*	Nein, nicht vorhanden	0
	Manchmal oder nur an einigen Orten oder nicht für alle genannten Gruppen verfügbar	2.5
	Ja, zu jeder Zeit und allen genannten Gruppen zugänglich	7.5
**Händehygiene und sanitäre Einrichtungen**		
**3. Sind funktionierende Händehygienestationen (d.h. alkoholische Desinfektionsmittellösungen oder Seife und Wasser sowie saubere Einweghandtücher) an allen Stellen der Patientenversorgung vorhanden?** *Wählen Sie bitte nur eine Antwort aus*	Nein, nicht vorhanden	0
	Ja, Händehygienestationen sind vorhanden, aber Verbrauchsmaterialien sind nicht zuverlässig verfügbar	2.5
	Ja, zuverlässig verfügbar	7.5
**4. Sind in Ihrem Krankenhaus/Ihrer Einrichtung ≥ 4 Toiletten[28] für ambulante Bereiche oder ≥ 1 pro 20 Benutzer für stationäre Bereiche verfügbar?** *Wählen Sie bitte nur eine Antwort aus*	Weniger als die benötigte Anzahl von Toiletten verfügbar und funktionierend	0
	Ausreichende Anzahl von Toiletten vorhanden, aber nicht alle funktionierend	2.5
	Ausreichende Anzahl von Toiletten vorhanden und funktionierend	7.5
**Stromversorgung, Lüftung und Reinigung**		
**5. Sind in Ihrem Krankenhaus/Ihrer Einrichtung Tag und Nacht ausreichend Strom/Energie für alle Anwendungen vorhanden (z.B. Pumpen und Kochen von Wasser, Sterilisation und Dekontamination, Abfallverbrennung oder alternative Abfalltechnologien, elektronische Medizinprodukte, allgemeine Beleuchtung von Bereichen, in denen Behandlungen stattfinden,**	Nein	0
	Ja, manchmal oder nur in einigen der genannten Bereiche	2.5

[27] Diese Komponente lässt sich detaillierter mit dem *WHO Water and sanitation for health facility improvement tool* (WASH-FIT) bewerten (http://www.who.int/water_sanitation_health/publications/water-and-sanitation-for-health-facility-improvement-tool/en/, Zugriff am 06. September 2018).
[28] Eine Toilette sollte über eine Tür verfügen, die von innen verschließbar und wenn die Toilette nicht verwendet wird, nicht verschlossen ist (bzw. ein Schlüssel zum Öffnen ist jederzeit verfügbar). Die Toiletten sollten keine relevanten Löcher bzw. Defekte aufweisen, nicht permanent verstopft sein und Wasser sollte in ausreichenden Mengen für den Spülvorgang zur Verfügung stehen. Die Sanitäranlagen befinden sich im Krankenhausbereich/im Bereich der Einrichtung und sollten dahingehend sauber sein, d.h. dass keine starke Verschmutzung mit Exkrementen oder Befall mit Insekten vorliegt.

**zur Gewährleistung einer sicheren Patientenversorgung sowie Beleuchtung von Toilettenanlagen und Duschen)?** *Wählen Sie bitte nur eine Antwort aus*	Ja, immer und in allen genannten Bereichen	5
**6. Ist eine funktionierende Be- und Entlüftung (natürlich oder mechanisch[29]) in den Bereichen der Patientenversorgung vorhanden?**	Nein	0
	Ja	5
**7. Existiert für Fußböden und horizontale Arbeitsflächen ein einsehbares Reinigungsprotokoll, das von den Reinigungskräften jeden Tag abgezeichnet wird?** *Wählen Sie bitte nur eine Antwort aus*	Kein Protokoll für zu reinigende Böden und horizontale Arbeitsflächen	0
	Protokoll existiert, aber wird nicht täglich ausgefüllt bzw. abgezeichnet oder ist veraltet	2.5
	Ja, Protokoll wird täglich ausgefüllt und abgezeichnet	5
**8. Sind geeignete und funktionierende Materialien für die Reinigung (z.B. Reinigungsmittel, Mops, Eimer usw.) vorhanden?** *Wählen Sie bitte nur eine Antwort aus*	Keine Materialien vorhanden	0
	Ja vorhanden, aber in schlechtem Zustand	2.5
	Ja, vorhanden und in einem guten Zustand	5
**Patientenunterbringung und persönliche Schutzausrüstung in der Krankenversorgung**		
**9. Haben Sie Einzelzimmer oder Räume für die Kohortierung[30] von Patienten mit ähnlichen Krankheitserregern (z.B. TBC, Masern, Cholera, Ebola, SARS)?[31]** *Wählen Sie bitte nur eine Antwort aus*	Nein	0
	Keine Einzelzimmer, aber geeignete Zimmer für die Patientenkohortierung	2.5
	Ja, Einzelzimmer sind verfügbar	7.5
**10. Steht eine persönliche Schutzausrüstung[32] jederzeit und in ausreichender Menge für alle Verwendungszwecke allen Mitarbeitern zur Verfügung?** *Wählen Sie bitte nur eine Antwort aus*	Nein	0
	Ja, aber nicht kontinuierlich in ausreichender Menge verfügbar	2.5
	Ja, kontinuierlich in ausreichender Menge verfügbar	7.5
**Medizinische Abfallwirtschaft und Abwasser**		
**11. Haben sie funktionierende Abfallsammelbehälter für nicht-infektiöse (allgemeine) Abfälle, infektiöse Abfälle und scharfe (spitze) Abfälle in unmittelbarer Nähe aller Abfallentstehungsorte?** *Wählen Sie bitte nur eine Antwort aus*	Keine Behälter bzw. keine separate Entsorgung für scharfe (spitze) Gegenstände	0
	Getrennte Behälter vorhanden, aber Deckel fehlen oder mehr als ¾ voll; nur zwei Behälter (anstelle von drei); oder Behälter an einigen, aber nicht allen, Abfallentstehungsorten	2.5
	Ja	5
**12. Ist eine Abfallgrube/umzäunte Mülldeponie oder eine städtische Abholung zur Beseitigung nicht infektiöser (nicht gefährlicher/allgemeiner) Abfälle vorhanden bzw. gewährleistet?** *Wählen Sie bitte nur eine Antwort aus*	Keine Abfallgrube oder andere Entsorgungsmethode vorhanden	0
	Abfallgrube vorhanden, aber unzureichende Größe; überfüllt oder nicht eingezäunt und verschlossen; oder unregelmäßige städtische Müllabholung	2.5

[29] Natürliche Belüftung: Außenluft, die von Naturkräften angetrieben wird (z.B. Wind), durch extra dafür installierte Öffnungen, wie Fenster, Türen, Solarschornsteine, Windtürme und Rieselventilatoren. Mechanische Belüftung: Luft, die von mechanischen Transportern angetrieben wird, die direkt in die Fenster oder Wände oder in Luftkanäle für die Zuführung von Luft in oder Abluft aus einem Raum installiert sind. Weitere Informationen unter http://www.who.int/water_sanitation_health/publications/natural_ventilation/en/, Zugriff am 06. August 2018.

[30] Kohortierungs-Stategien sollten auf einer Risikobewertung des Hygiene-/IPC-Teams basieren.

[31] Unterdruckbelüftung in Isolationsräumen kann zur Verhinderung der Übertragung bestimmter Organismen (z.B. TBC) erforderlich sein.

[32] Persönliche Schutzausrüstung: Einmalhandschuhe (keimarm bzw. steril), Mund-Nasen-Schutz, Schutzbrille oder Gesichtsschutz und Schutzkittel stellen die essenzielle persönliche Schutzausrüstung dar. Atemmasken und Schürzen sollten ebenfalls in adäquaten Mengen für den Bedarfsfall vorgehalten werden.

	Ja	5
**13. Ist eine Müllverbrennungsanlage oder eine alternative Technologie (entweder direkt vor Ort oder durch ein geeignetes externes lizensiertes Unternehmen durchgeführt) für die Behandlung von infektiösen und scharfen (spitzen) Abfällen (z.B. ein Autoklav) funktionsfähig und mit ausreichender Kapazität vorhanden?** *Wählen Sie bitte nur eine Antwort aus*	Nein, keine vorhanden	0
	Vorhanden, aber nicht funktionsfähig	2.5
	Ja	5
**14. Wird das Abwasser aufbereitet (z.B. Kläranlage oder Klärgrube, gefolgt von Entwässerungsgrube) oder in ein funktionierendes Abwassersystem geleitet?** *Wählen Sie bitte nur eine Antwort aus*	Nein, nicht vorhanden	0
	Ja, aber nicht verlässlich funktionierend	2.5
	Ja, und verlässlich funktionierend	5
**Dekontamination und Sterilisation**		
**15. Verfügt Ihr Krankenhaus/Ihre Einrichtung über einen eigenen Aufbereitungsbereich und/oder eine sterile Versorgungsabteilung (ZSVA/AEMP) (entweder direkt vor Ort oder durch ein geeignetes externes lizensiertes Unternehmen durchgeführt) für die Aufbereitung und Sterilisation von Medizinprodukten und anderen Gegenständen/Ausrüstung?** *Wählen Sie bitte nur eine Antwort aus*	Nein, nicht vorhanden	0
	Ja, aber nicht verlässlich funktionierend	2.5
	Ja, und verlässlich funktionierend	5
**16. Stehen sterile und desinfizierte Geräte zum Gebrauch zur Verfügung?** *Wählen Sie bitte nur eine Antwort aus*	Nein, im Durchschnitt < 5 Tage pro Woche	0
	Ja, im Durchschnitt ≥ 5 Tage pro Woche oder jeden Tag, aber nicht in ausreichender Menge	2.5
	Ja, jeden Tag und in ausreichender Menge vorhanden	5
**17. Stehen im Bedarfsfall Einmalprodukte (z.B. Sicherheitskanülen, Untersuchungshandschuhe) zur Verfügung?** *Wählen Sie bitte nur eine Antwort aus*	Nein, nicht vorhanden	0
	Ja, aber nur manchmal verfügbar	2.5
	Ja, und kontinuierlich verfügbar	5
**Zwischensumme**		**/100**

Interpretation Ihrer Antworten: ein dreistufiger Prozess

1. Bitte fügen Sie Ihre Punktzahlen ein

	**Punktzahl**
**Themengebiet/Kernkomponente**	**Punktzahl - Zwischensumme**
**1.** Hygiene-/IPC-Programm	
**2.** Hygiene-/IPC-Leitlinien	
**3.** Hygiene-/IPC-Ausbildung und Schulung	
**4.** Surveillance von nosokomialen Infektionen	
**5.** Multimodale Strategien	
**6.** Monitoring/Audits	
**7.** Arbeitsaufwand, Personal und Bettenbelegung	
**8.** Strukturgestaltung, Materialien und Ausstattung	
**Gesamt**	**/800**

2. Bestimmen Sie die zugewiesene „Hygiene-/IPC-Kategorie" für Ihr Krankenhaus/Ihre Einrichtung. Nehmen Sie dazu die Gesamtpunktzahl aus Schritt 1.

**Gesamtpunktzahl**	**Hygiene-/IPC-Kategorie**
0 – 200	Unzureichend
201 - 400	Grundlegend
401 - 600	Intermediär
601 - 800	Fortgeschritten

3. **Überprüfen Sie die Ergebnisse des IPCAF und erstellen Sie einen Aktionsplan**
Überprüfen Sie die von dieser Bewertung identifizierten Bereiche, die einer Verbesserung bedürfen, und erstellen Sie einen Aktions-/Maßnahmenplan, der diese angehen soll (als Referenz (inkl. Listen mit Verbesserungswerkzeugen, Anleitungen, Formblätter, Tipps und Beispielen aus der ganzen Welt): *Interim practical manual* der WHO zur Implementierung der WHO-Guidelines zur Infektionsprävention und –kontrolle unter http://www.who.int/infection-prevention/tools/core-components/en/, Zugriff am 06. August 2018). Behalten Sie eine Kopie von diesem Erhebungsbogen, um die aktuelle Bewertung mit Wiederholungen in der Zukunft zu vergleichen.
