# Supplementary material for: Evaluating infection prevention and control programs in Austrian acute care hospitals using the WHO Infection Prevention and Control Assessment Framework
Source: Antimicrob Resist Infect Control. 2020 Jun 22;9:92. doi: 10.1186/s13756-020-00761-2 (PMC7309981; doi:10.1186/s13756-020-00761-2)
Supplement: Supplementary file 2 — Additional file 2: Table e1. Structural characteristics of 127 Austrian acute care hospitals invited to participate in the WHO Infection Prevention and Control Assessment Framework (IPCAF). [file 13756_2020_761_MOESM2_ESM.pdf]

Table e1 Structural characteristics of 127 Austrian acute care hospitals invited to participate in the WHO Infection Prevention and Control Assessment Framework (IPCAF)

**Parameter**	**Variable** or **Group**	**Value** or **Number (percentage)**
Hospital size (i.e. number of hospital beds)	First quartile	152
	Median	224
	Third quartile	425
Hospital type	Primary care	49 (38.6)
	Secondary care	24 (18.9)
	Tertiary care	26 (20.5)
	Maximum care (incl. university hospitals)	10 (7.9)
	Specialized hospital	9 (7.1)
	Other/Unknown	9 (7.1)
Hospital ownership	Public (not further specified)	99 (78.0)
	Private (not further specified)	12 (9.4)
	Ecclesiastical	11 (8.7)
	Other/Unknown	5 (3.9)
